# Laser Capture Microdissected Mucosa versus Whole Tissue Specimens for Assessment of Radiation-Induced Dynamic Molecular and Pathway Changes in the Small Intestine

**DOI:** 10.1371/journal.pone.0053711

**Published:** 2013-01-14

**Authors:** Junying Zheng, Sarita Garg, Junru Wang, David S. Loose, Martin Hauer-Jensen

**Affiliations:** 1 Division of Radiation Health, Department of Pharmaceutical Sciences, University of Arkansas for Medical Sciences, Little Rock, Arkansas, United States of America; 2 Integrative Biology and Pharmacology, UTHealth Medical School, Houston, Texas, United States of America; 3 Surgical Service, Central Arkansas Veterans Healthcare System, Little Rock, Arkansas, United States of America; Vanderbilt University, United States of Amerifca

## Abstract

**Background:**

The intestinal mucosa is the compartment that sustains the most severe injury in response to radiation and is therefore of primary interest. The use of whole gut extracts for analysis of gene expression may confound important changes in the mucosa. On the other hand, laser capture microdissection (LCM) is hampered by the unstable nature of RNA and by a more complicated collection process. This study assessed, in parallel samples from a validated radiation model, the indications for use of LCM for intestinal gene expression analysis.

**Methodology/Principal Findings:**

RNA was extracted from mouse whole intestine and from mucosa by LCM at baseline and 4 h, 24 h, and 3.5 d after total body irradiation and subjected to microarray analysis. Among mucosal genes that were altered > = 2-fold, less than 7% were present in the whole gut at 4 and 24 h, and 25% at 3.5 d. As expected, pathway analysis of mucosal LCM samples showed that radiation activated the coagulation system, lymphocyte apoptosis, and tight junction signaling, and caused extensive up-regulation of cell cycle and DNA damage repair pathways. Using similar stringent criteria, regulation of these pathways, with exception of the p53 pathway, was undetectable in the whole gut. Radiation induced a dramatic increase of caspase14 and ectodysplasin A2 receptor (Eda2r), a TNFα receptor, in both types of samples.

**Conclusions/Significance:**

LCM-isolated mucosal specimens should be used to study cellular injury, cell cycle control, and DNA damage repair pathways. The remarkable increase of caspase14 and Eda2r suggests a novel role for these genes in regulating intestinal radiation injury. Comparative gene expression data from complex tissues should be interpreted with caution.

## Introduction

The intestine is a dose limiting organ during radiation therapy of abdominal cancers and a critical determinant of survival in radiological emergency situations. This is mainly due to the radiosensitivity of its rapidly proliferating crypt epithelium. The intestinal mucosa invariably sustains the most severe injury and undergoes the most change after exposure to ionizing radiation [1]–[3] and is therefore the compartment of primary interest.

Methods such as *in situ* hybridization and immunohistochemistry have provided substantial information about the molecular and cellular events that occur in the bowel mucosa in response to ionizing irradiation. However, for analysis of differential gene expression patterns and pathways regulated during the development of radiation toxicity, more powerful methods such as expression arrays must be used.

The isolation of RNA from whole tissue is relatively quick, easy, and may reveal important information about differential gene expression patterns. However, the utility of analysis performed on whole gut RNA extracts is questionable because it is hampered both by the structural complexity of the intestine, as well as by the relative change in structure that the mucosa undergoes during the process of development of radiation toxicity. In contrast, RNA isolated by laser capture microdissection (LCM) can be used to isolate relatively pure cell populations or tissue segments of interest from fixed tissue sections, thereby avoiding both of the problems associated with whole tissue RNA extracts [4].

LCM is widely used for gene expression analysis in both basic research and clinical applications [5]–[8]. However, although RNA extracted from LCM samples is intuitively preferable to whole tissue RNA, it has several disadvantages. First, due to the unstable nature of intestinal RNA, it is more challenging to obtain sufficiently high quality RNA from LCM samples. Second, the RNA isolation process is more cumbersome, lengthy and expensive. Lastly, the preparation of tissues and the process of performing the LCM are much more time-consuming. For these reasons, it is important to establish if and for what pathways LCM is necessary.

Here, a well-defined mouse model of total body radiation was used to compare the transcriptional profiles of LCM-separated mucosal and whole gut specimens obtained in parallel. Our results clearly revealed that it is important to use LCM for analysis of pathways related to cellular injury, cell cycle control, and DNA damage repair pathways, all pathways that are central in the radiation response. Only genes where there are substantial differences in expression levels, such as certain apoptotic and cytokine signaling pathways, can be safely analyzed in whole gut RNA extracts.

## Materials and Methods

### Animals

The experimental protocol was reviewed and approved by the University of Arkansas for Medical Sciences and the Central Arkansas Veterans Healthcare System's (CAVHS) Institutional Animal Care and Use Committees (IACUC).

Experiments were carried out in a total of 40 random-bred male CD2F1 mice (Harlan Sprague Dawley, Indianapolis, IN), 6–7 weeks old on arrival and with an initial body weight of 22–25 g. Animals were housed in conventional cages under standardized conditions with controlled temperature and humidity and a 12–12-h day-night light cycle. Animals had free access to water and chow (Harlan Teklad laboratory diet 7012, Purina Mills, St. Louis, MO).

### Irradiation

Total body γ irradiation was performed as described before [9]. Briefly, after confirmation of dose uniformity by thermoluminescence dosimetry, irradiation was performed with a Shepherd Mark I, model 25, 137Cs irradiator (J. L. Shepherd & Associates, San Fernando, CA). During irradiation, the animals were held in well-ventilated custom-made Plexiglas restrainers on a turntable rotating at 5 revolutions per minute. The average dose rate was 1.35 Gy per minute. Mice were exposed to sublethal (8.0 Gy) single dose total body irradiation (TBI). Groups of 8–10 mice were killed humanely at set times after radiation [0 h (no radiation), 4 h, 24 h, 3.5 days,]. Samples from individual mice were analyzed throughout, i.e., without pooling.

### Laser capture microdissection

**Figure 1:**
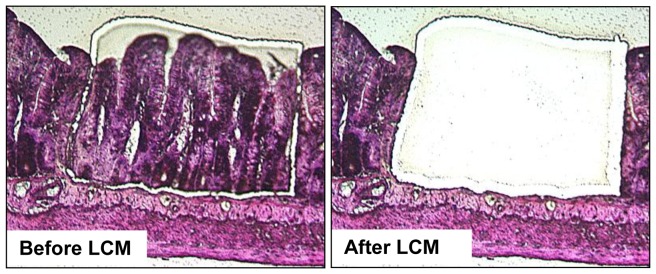
Images of HE stained jejunum sections (20×) before and after Laser Capture Microdissection.

Collection of intestinal mucosa was performed by LCM as shown in Figure 1. Briefly, 10-µM thick sections of the jejunum were cut from paraffin embedded intestinal tissue and mounted onto cross-linked polyethylene (PEN) foil attached to a glass slide (Leica, Wetzlar, Germany) to facilitate removal of the selected specimen from the slide to the PCR tube. The slides were then further processed for hematoxylin staining. A Leica AS LMD microdissection system (Leica), which utilizes a pulsed UV laser that melts or cuts the foil film, was used to capture intestinal mucosal tissue. Mucosa was outlined by free-hand tracing and was cut from the slide by the laser and collected into the cap of a 0.2 ml PCR tube containing 30 µl of lysis buffer. All samples were stored at −80°C until processed for RNA as one batch.

### RNA extraction from LCM dissected mucosa and whole gut samples

Mucosal RNA was extracted from the LCM-dissected jejunum using the RNAqueous-Micro kit (Ambion, Austin, TX) specifically designed to recover small amounts of RNA from LCM samples.

Whole gut RNA was extracted from jejunum collected at different time points following irradiation. Tissues were snap frozen in liquid nitrogen and stored at −80°C. Frozen tissue samples were homogenized in Ultraspec RNA reagent (Biotecx Laboratories, Houston, TX), according to the manufacturer's instructions. Two µg of RNA were kept for microarray analysis (including quality control analysis) and 2 µg were used for real time PCR.

DNase-treated RNA was quantitated using the Rediplate 96 Ribogreen RNA quantitation kit (Invitrogen, Carlsbad, CA).

### DNA microarray analysis

RNA samples from LCM dissected mucosa and whole gut were screened by checking their integrity and genomic DNA contamination. Selected RNA samples were processed for microarray analysis using Illumina one-color MouseWG-6_V2 chip. The sample size for LCM and for whole gut microarray was N = 4/time point.

Microarray data was analyzed by Genespring GX 11.0 (Agilent Technologies, CA). Raw data were log2 transformed and then normalized to the 75th percentile of all values on a chip. Student's T-test was used to examine for differentially expressed genes. A list of genes with ≥2.0-fold change was generated first and tested by the Benjamini-Hochberg Multiple Testing Correction procedure. Significant genes were selected by a cut-off of p<0.05 and fold change > = 2.0.

### Ingenuity Pathway Analysis (IPA)

The selected genes were subsequently analyzed using IPA 5.0 (Ingenuity Systems Inc., CA). Pathways which were predicted to be influenced by the differentially expressed genes were ranked in order of significance and further analyzed by overlaying with function, disease and canonical pathways. An overlap p-value<0.05 indicates a statistically significant, non-random association between a set of genes in the dataset and a related function. The p-value of overlap is calculated by the Fisher's Exact Test.

### Verification of Gene Expression Using Real-Time RT-PCR

Verification of gene expression was carried out using different sets of total RNA samples from those that were sent for microarray. For each RNA sample, 1 µg was used as the template for cDNA synthesis by using the High Capacity cDNA reverse transcription kit (Applied Biosystems, Foster City, CA) according to the protocol provided by the manufacturer. Steady-state mRNA levels were measured with real-time quantitative PCR using the following Applied Biosystems predesigned Taqman gene expression assays: Casp14, Mbl2, Mcpt1, Eda2r, CCL-9 and IL-33. PCR amplification and detection were carried out on an ABI prism 7000 Detection System (Applied Biosystems). Transcript levels were normalized to eukaryotic 18S rRNA and calculated relative to control, unirradiated jejunum, using the standard ΔΔCt method.

### TUNEL staining

5 µm sections were mounted on glass slides. TUNEL assay was performed using the Dead End fluorometric TUNEL kit (Promega, Madison, WI) following the manufacturer's instruction. Image acquisition was performed on a Zeiss Imager Z1 Epifluorescence Microscope (Zeiss, Goettingen, Germany).

### Immunohistochemistry (IHC)

5 µm sections were mounted on slides, deparaffinized in xylene, and rehydrated in graded solutions of ethanol. After blocking with 5% normal serum, sections were incubated with primary antibody overnight at 4°C, followed with Alexa Fluor conjugated secondary antibody incubation (Molecular Probes, Eugene, OR; 1∶400) for 1 h at room temperature. Slides were mounted with Vectashield Mounting Medium with DAPI for nuclear staining (Vector Laboratories, Burlingame, CA). Image acquisition and intensity measurements were performed on a Zeiss Imager Z1 Epifluorescence Microscope (Zeiss, Goettingen, Germany).

### Statistics

Data are presented as mean ± standard deviation. Statistical analysis was performed by t-test. p<0.05 was considered to be significant. For the microarray, a list of genes with > = 2-fold change was generated by t-test and filtered by the “Benjamini and Hochberg false discovery test”. Genes with a false discovery of less than 0.05 were used to for the final list of differentially expressed genes. For IPA, the p-value of overlap is calculated by the Fisher's Exact Test.

## Results

### TUNEL staining of mouse intestine at 0, 4 h, 24 h, and 3.5 day after radiation

**Figure 2:**
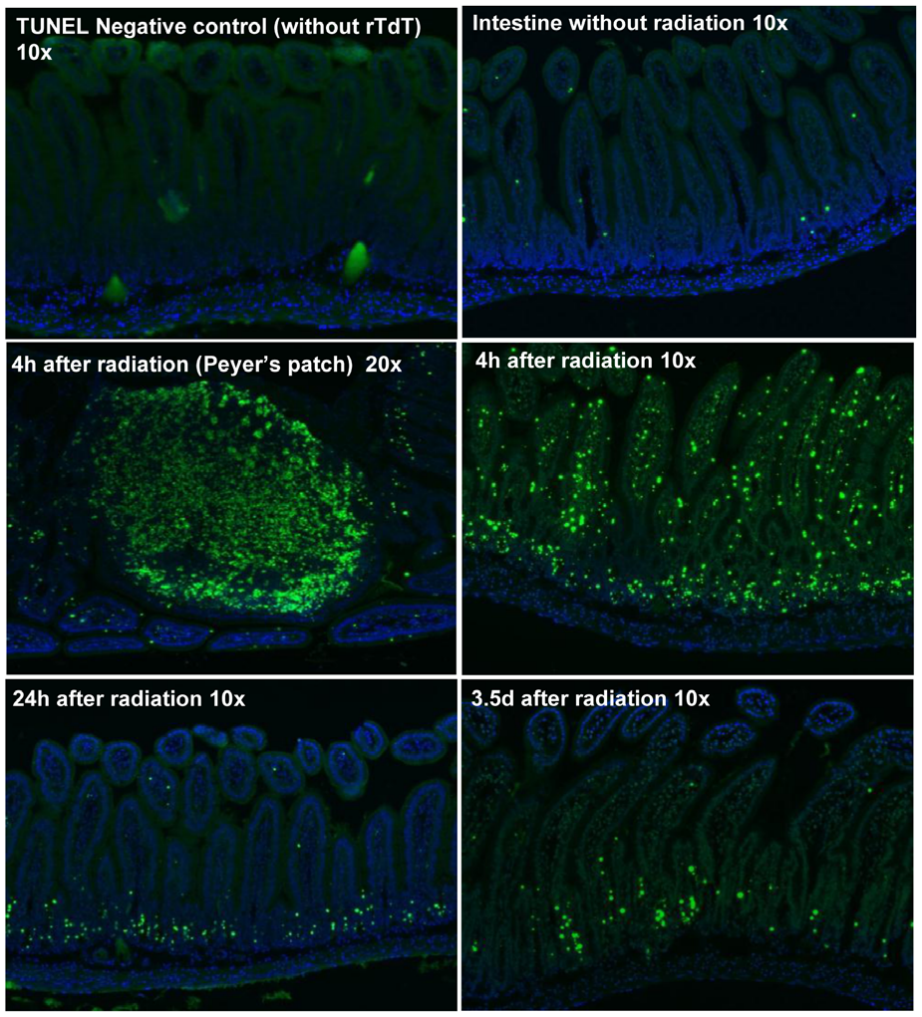
DNA damage in mouse small intestine at baseline and different time points. TUNEL staining to demonstrate DNA damage in mouse small intestine at baseline and 4 h, 24 h, and 3.5 d after irradiation. Green: TUNEL positive, Blue: DAPI. 10×/20×: the magnifying power of the objectives. rTdT: recombinant Terminal Deoxynucleotidyl Transferase enzyme.

To verify radiation-induced cellular injury/death, TUNEL staining was applied on the intestinal tissue at each time point. Figure 2 demonstrates radiation- induced TUNEL positive nuclei in intestinal mucosa at 0 h to 3.5 d post radiation. Radiation induced massive nuclear DNA damage at 4 h in mucosa that was gradually reduced at 24 h and 3.5 d. TUNEL staining also demonstrated substantial lymphocyte nuclear DNA damage in Peyer's patches 4 h after radiation [10].

### Microarray Analysis

**Figure 3:**
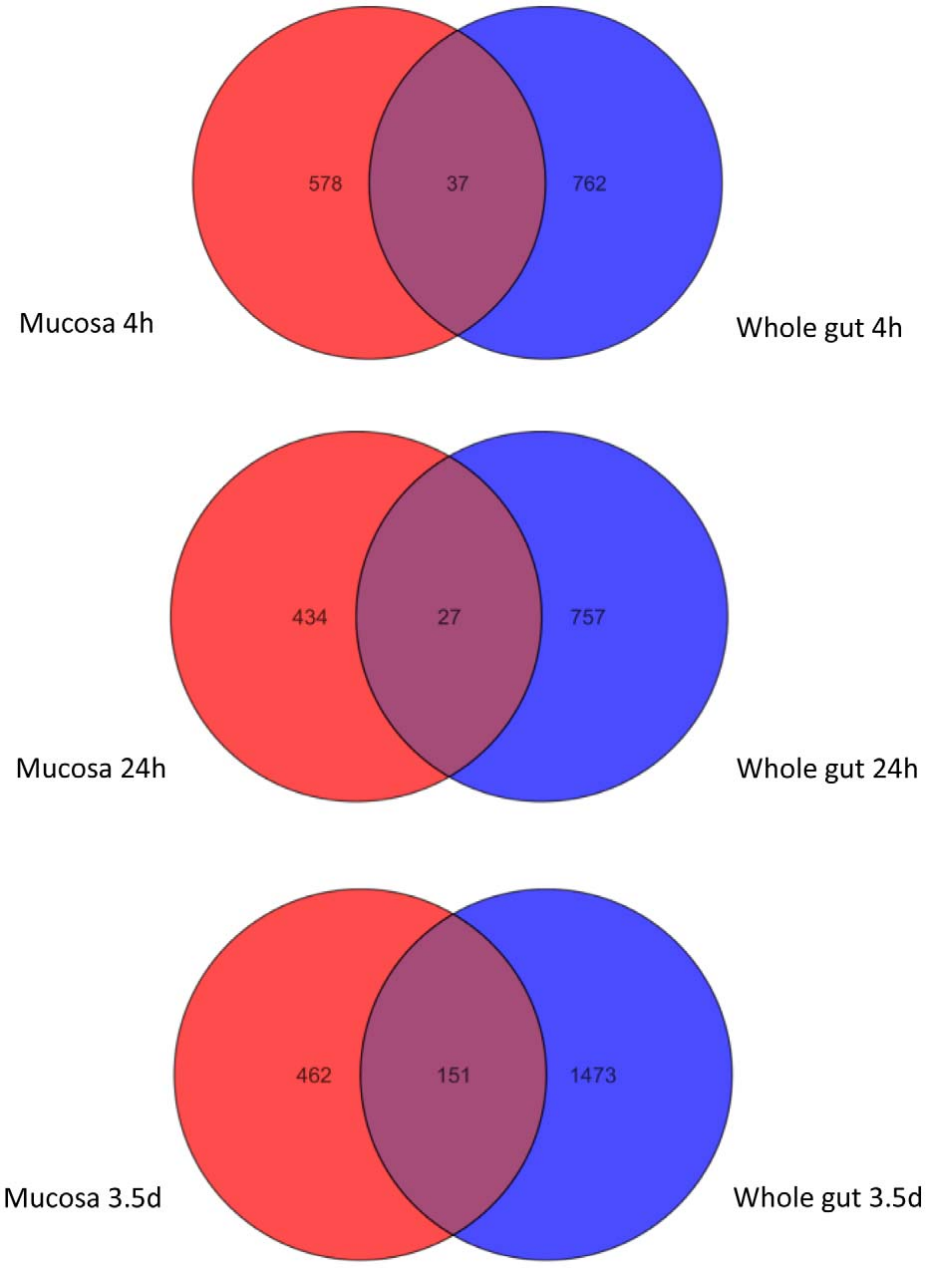
Venn diagram demonstrating radiation- induced differential transcriptional profiles in mucosa and whole gut.

The gene list with a cut-off of 2-fold and p-value<0.05 was generated at each time point. In LCM samples, the number of genes that were altered ≥2-fold was 615 at 4 h, 461 at 24 h, and 613 at 3.5 d post radiation. In the whole gut, the number of genes that altered ≥2-fold was 799 at 4 h, 784 at 24 h, and 1624 at 3.5 d post radiation. Venn diagrams (Figure 3) demonstrated an overlap of the changed genes in LCM and whole gut at each time point. Among the mucosal genes that were altered > = 2-fold, less than 7% was changed in the whole gut at 4 and 24 h, and 25% at 3.5 d.

### Verification of Gene Expression Using Real-Time RT-PCR

**Figure 4:**
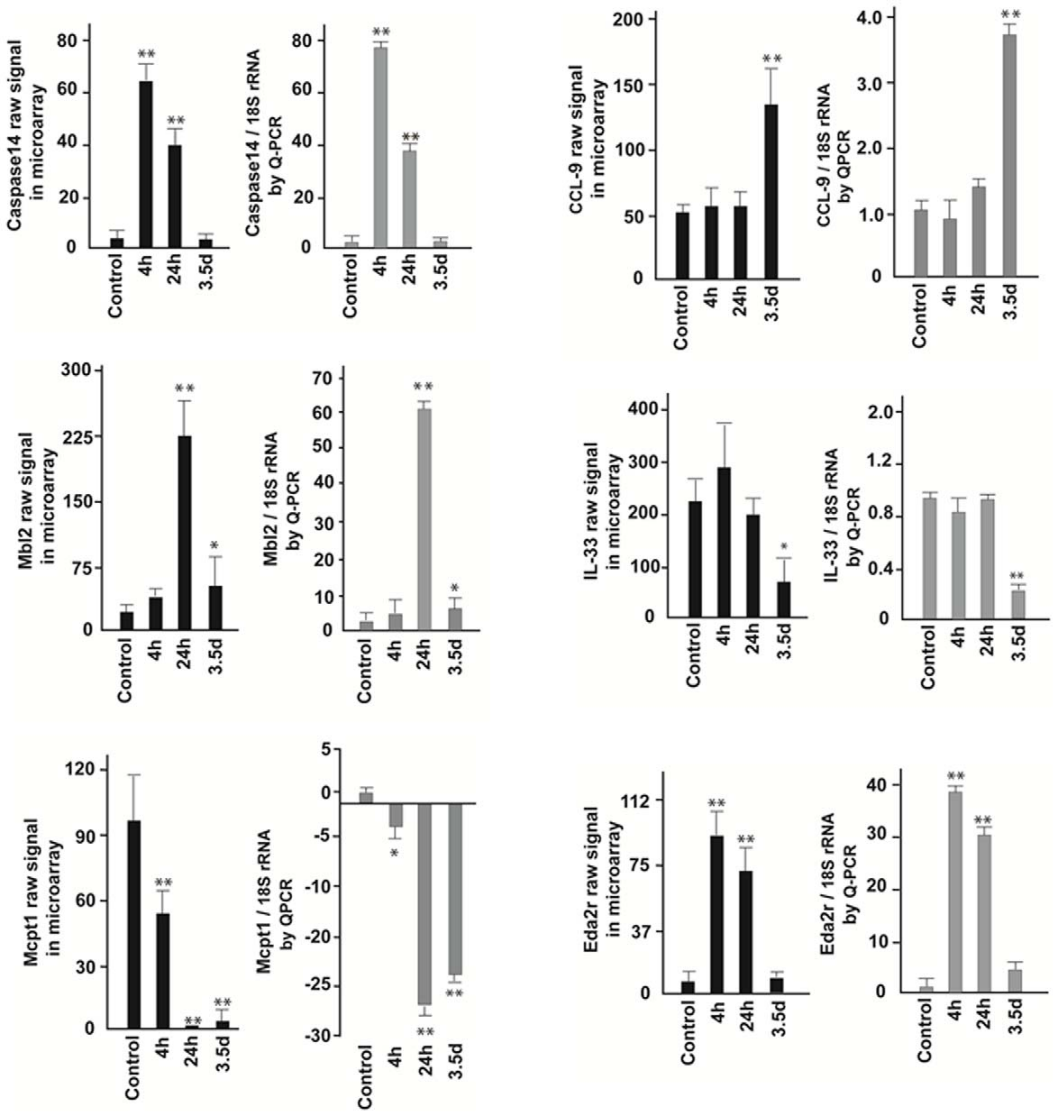
Validation of the selected genes in the whole gut by real-time RT-PCR. The left panels (black bars) demonstrate expression of the selected genes at different time points plotted by raw signals from the microarray. The right panels (gray bars) show the relative expression of the same selected genes normalized by 18S rRNA at different time points by real-time RT-PCR. The opposite direction of Mcpt1 demonstrates its down-regulation after radiation.

The change of selected genes of Casp14, Mbl2, Mcpt1, CCL-9, IL-33 and Eda2r at 4 different time points were validated by real-time RT-PCR using Applied Biosystems predesigned Taqman gene expression assays (Figure 4). The left panels (black bars) demonstrate the expression of the selected genes at different time points in the whole gut plotted by raw mRNA signals from the microarray. The right panels show the relative expression of the gene normalized by 18SrRNA at different time points in the whole gut by real-time RT-PCR.

### Comparative IPA analysis in mucosa and whole gut

#### Activation of the coagulation system, cellular injury, and DNA damage repair pathways

**Figure 5:**
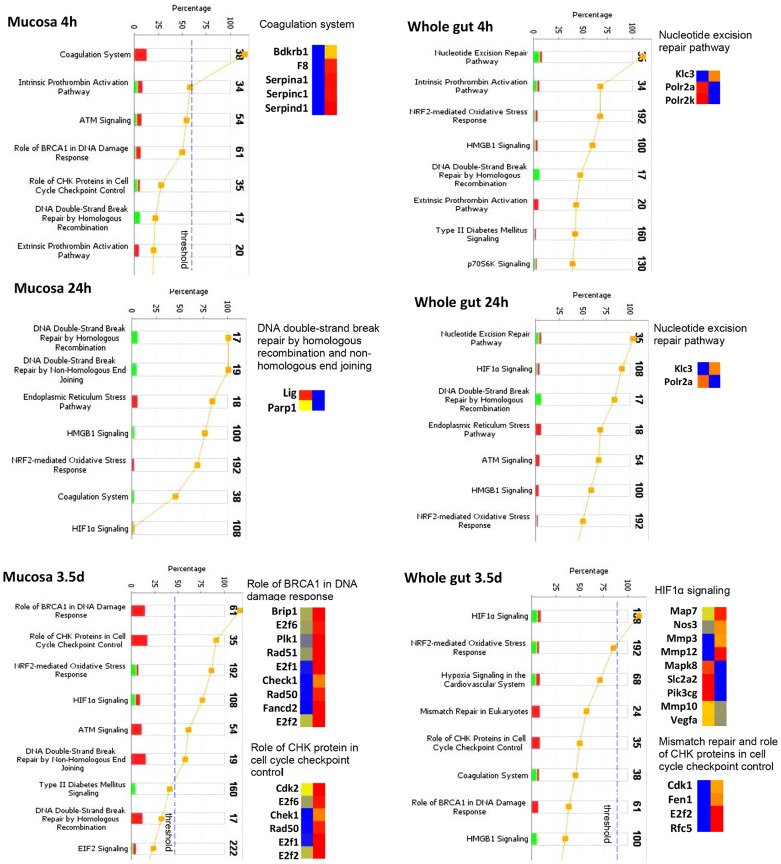
Activation of cellular injury and DNA damage repair pathways. Stacked bar charts demonstrate IPA-generated activated cellular injury and DNA damage repair pathways in mucosa and whole gut at 4 h, 24 h, and 3.5 d after irradiation. The height of the bars indicates the percentage of genes that changed in the particular pathway. Red bar: up-regulated. Green bar: down-regulated. Pathways with p-value (yellow dot) above the threshold (dashed line) are significantly activated. Heatmaps demonstrate the change of the genes in the selected signaling pathway before (left column) and after radiation (right column). Blue: decreased, red: increased.

The lists of altered genes were imported into IPA for pathway analysis. Figure 5 demonstrates activation of pathways mediating cellular injury and DNA damage repair at different time points in mucosa and whole gut. In mucosa, at 4 h after radiation exposure, Bdkrb1, F8, Serpina1, Serpinc1 and Serpind1 were upregulated, consistent with activation of the coagulation system. The activated coagulation system was significantly suppressed at 24 h and 3.5 d. At 3.5 d, there was extensive up-regulation of DNA damage repair pathways such as BRCA1, CHK, and ATM signaling as well as homologous and non-homologous DNA double-strand break repair signaling.

In the whole gut, there were no significantly activated cellular injury pathways at 4 and 24 h. At 3.5 d, up-regulation of DNA damage repair was not significant. Instead, there was a down-regulated HIF1α signaling.

#### Activation of pathways involved in cell cycle control

**Figure 6:**
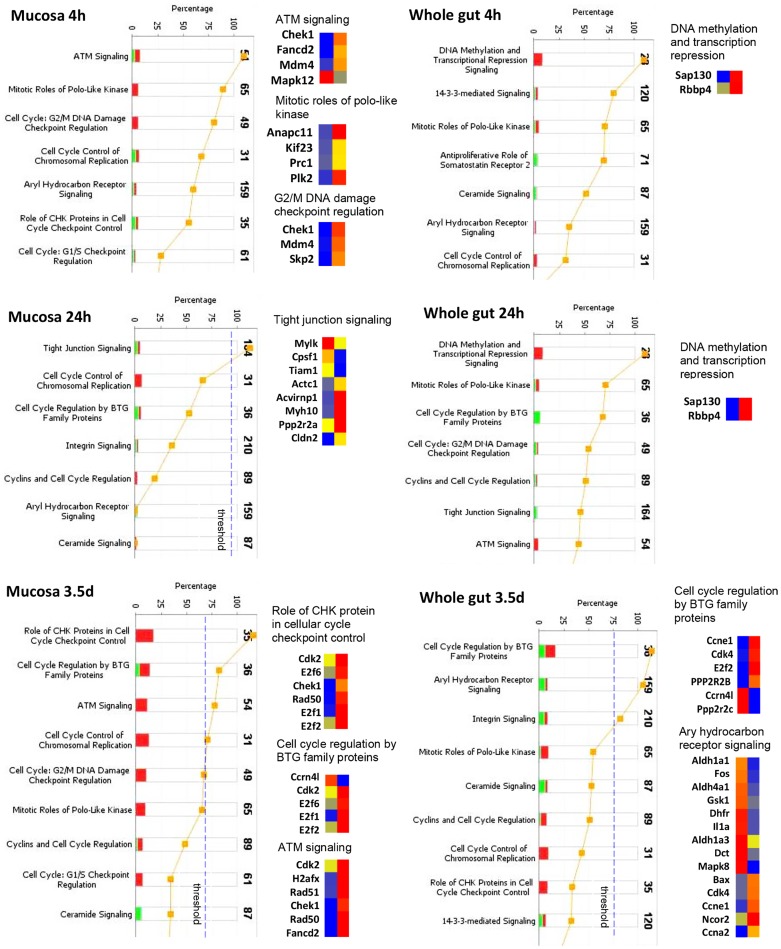
Activation of cell cycle control pathways in mucosa and whole gut. Stacked bar charts demonstrate IPA-generated activated cell cycle control pathways in mucosa and whole gut at 4 h, 24 h, and 3.5 d after irradiation. The height of the bars indicates the percentage of genes that changed in the particular pathway. Red bar: up-regulated. Green bar: down-regulated. Pathways with p-value (yellow dot) above the threshold (dashed line) are significantly activated. Heatmaps demonstrate the change of the genes in the selected signaling pathway before (left column) and after radiation (right column). Blue: decreased, red: increased.

Corresponding to cellular injury and DNA damage repair, Figure 6 demonstrates radiation induced activation of cell cycle control pathways. In mucosa, no significant changes of cell cycle control pathways were observed at 4 h. At 24 h, tight junction signaling was activated suggesting a response to the break of the epithelial barrier. At 3.5 days, there was wide up-regulation of various pathways regulating cell cycle such as CHK, BTG, ATM, and G2/M DNA damage checkpoint regulation signaling.

In the whole gut, there was no significantly activated pathway at 4 and 24 h. At 3.5 d, among the highly up-regulated pathways in the mucosa, only BTG was significantly changed (Figure 6).

#### Activation of apoptosis pathways

**Figure 7:**
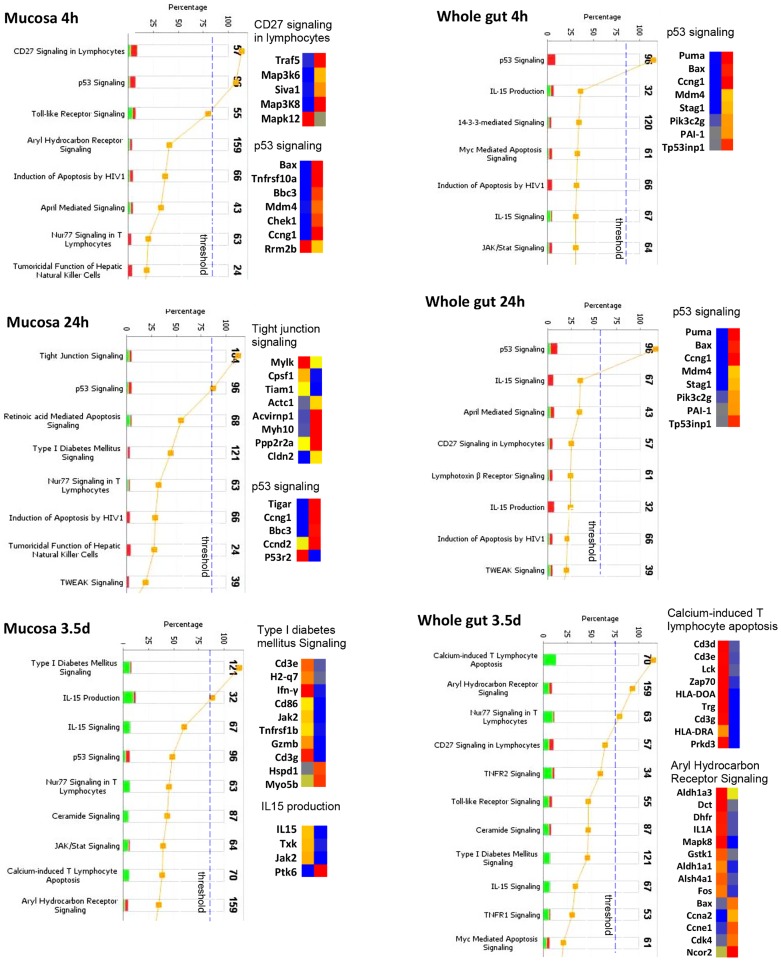
Activation of apoptosis pathways in mucosa and whole gut. Stacked bar charts demonstrate IPA-generated activated apoptosis pathways in mucosa and whole gut at 4 h, 24 h, and 3.5 d after irradiation. The height of the bars indicates the percentage of genes that changed in the particular pathway. Red bar: up-regulated. Green bar: down-regulated. Pathways with p-value (yellow dot) above the threshold (dashed line) are significantly activated. Heatmaps demonstrate the change of the genes in the selected signaling pathway before (left column) and after radiation (right column). Blue: decreased, red: increased.

As a rapidly renewing tissue, the intestine undergoes extensive cell death in response to radiation. To study the global impact of gene expression profiles on cell death in mucosa and whole gut, regulation of apoptosis pathways was analyzed by IPA. Figure 7 demonstrates a dynamic change of apoptosis pathway in mucosa and whole gut at 4 h, 24 h and 3.5 d. In the mucosa, at 4 h after irradiation, there were two significantly up-regulated pathways, including lymphocyte CD27 signaling and p53 signaling. At 24 h, disrupted tight junction signaling was observed. At 3.5 d, there was wide down-regulation of apoptosis pathways, including type I diabetes mellitus signaling, IL-15 production and IL-15 signaling.

In whole gut, p53 signaling was significantly up-regulated at both 4 h and 24 h. At 3.5 day, there was wide down regulation of various genes involved in apoptosis such as Calcium-induced T lymphocyte apoptosis signaling, Aryl hydrocarbon receptor signaling, Nur77 signaling in lymphocytes, and CD27 signaling in lymphocytes.

### Immunohistochemistry (IHC) of caspase-14

**Figure 8:**
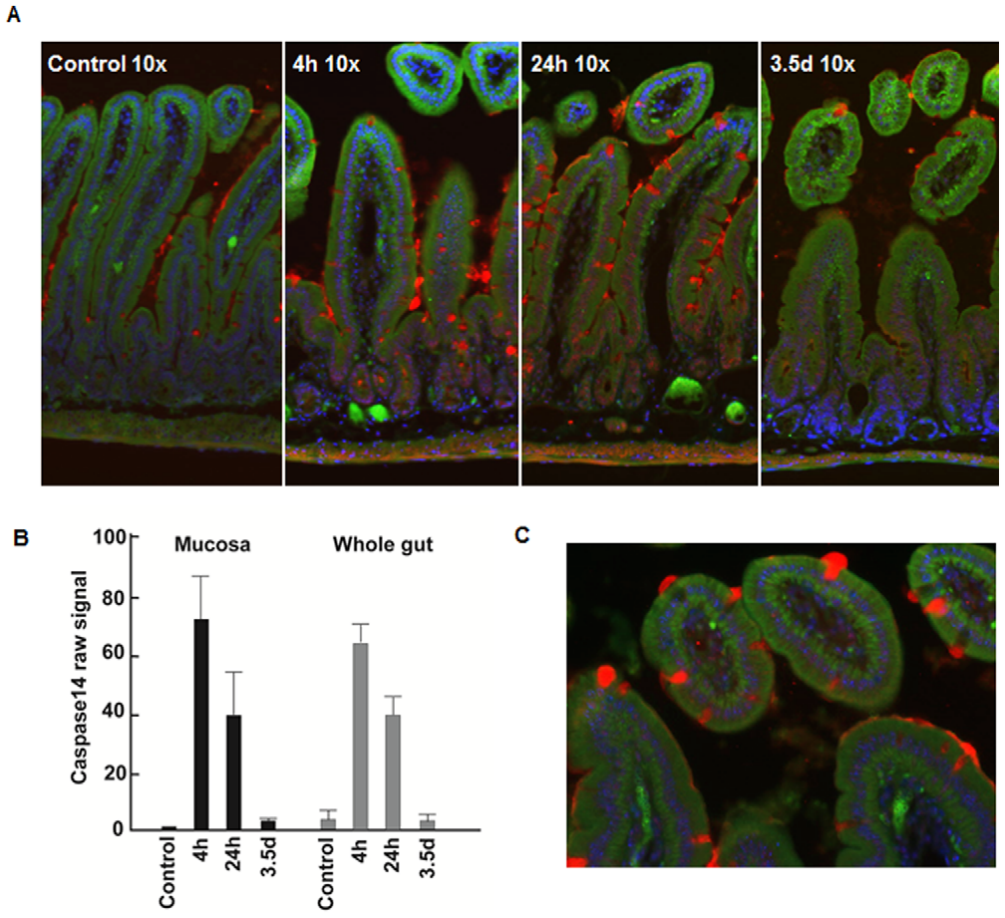
Expression of Caspase14 mRNA and protein in the small intestines. **A**: Immunohistochemistry demonstrating the expression of Caspase14 in goblet cells in baseline (control) and irradiated intestine at 4 h, 24 h and 3.5 d post irradiation. **B**: Expression of Caspase14 mRNA in mucosa and whole gut tissue plotted by the raw signal from microarray. **C**: A higher magnification power demonstrating goblet cells secreting Caspase14 at 24 h post irradiation. Red: Caspase14; Green: E-cadherin; Blue: DAPI.

Gene microarray revealed that radiation induced a striking increase of casp-14, both in LCM samples from the intestinal mucosa and in samples from the whole gut. For example, while expression of casp14 mRNA was very low under normal conditions in mucosa, it increased 67-fold at 4 h, 33-fold at 24 h and then dropped to baseline levels at 3.5 d (Figure 4, Figure 8). IHC verified the increased casp14 protein at 4 and 24 h, while at 3.5 d, the expression of casp-14 protein had decreased to baseline levels. IHC staining also showed that casp14 secretion was localized to goblet cells which were largely distributed on the surface of mucosal villi, although there was also some positive casp14 signal in the crypt area (Figure 8).

## Discussion

LCM is a technique by which pure cell populations or tissue segments of interest can be procured from frozen or fixed tissue sections. Since the first development of LCM in 1996, it has become widely used in the basic research and clinical areas for the study of *in vivo* cell alterations during disease development. As the intestinal mucosa manifests the most severe injury among the compartments of the small intestinal wall, studying changes in gene expression in the mucosa can provide important information about the mechanisms of radiation injury. Unfortunately, because of the extremely unstable nature of intestinal RNA, preparing RNA from mucosal LCM samples is cumbersome, expensive and the preparation of tissues and process of LCM rather time-consuming. This study used a well-defined mouse model of total body irradiation to perform a rigorous comparison of the transcriptional profiles of parallel LCM-separated mucosal and whole gut specimens at various times after irradiation. We found that radiation induced strong activation of pathways mediating cellular injury, lymphocyte apoptosis, tight junction, cell cycle control and DNA damage repair at different time points in mucosa but, with exception of the p53 pathway, not in the whole gut. These results, which to our knowledge are the first from a systematic, rigorous comparison of LCM and whole tissue RNA extracts, suggest that obtaining mucosal samples is essential for the study of these critical signaling pathways. In addition, we report that the newly identified genes of Casp14 and Eda2r could provide novel research objectives and offer new therapeutic strategies for intestinal radiation injury.

A single dose (8.0 Gy) of total body γ radiation to CD2F1 mice did not cause significant 30-day lethality but considerable histological damage and, as shown by TUNEL staining coincident with upregulation of CD27 signaling, a wave of apoptosis in mucosa [10], [11]. Pathway analysis revealed a strong activation of the coagulation system, indicative of damage to the endothelial lining of microvessels. The up-regulation of genes coding anticoagulant factors such as Serpinc1 (ATIII) and Serpind1 (heparin cofactor II) may suggest a link of activated anticoagulant factors to intestinal bleeding [12]–[14]. The increased expression of the procoagulant factors F8 [15] and Bdkrb1 [16], [17] as well as an inhibitor of a wide variety of proteases Serpina1 (alpha-1 antiproteinase) [18] could be explained as a compensatory response to achieve a balanced hemostasis and protect the tissue from degradation. The activation of coagulation system at 4 h was coincident with the maximum occurrence of TUNEL positive nuclei, consistent with a cause-effect relationship between endothelial dysfunction and mucosal cell death [19].

At 24 h post radiation, the most significantly changed pathways were tight junction and p53 signaling pathways. The disrupted tight junction signaling in mucosa could possibly explain a response to the breakdown of the integrity of epithelial tight junction. There was significant up-regulation of p53 signaling both in the mucosa and the whole gut. The up-regulated p53 signaling in mucosa and whole gut at 24 h while TUNEL positive nuclei were significantly decreased at this time could repudiate the direct link of p53 to cell death in the intestine, as documented in other models [20]–[23]. No significant activation on cell cycle control and DNA damage signaling was observed at this point.

A broad up-regulation of cell cycle control and DNA damage repair pathways occurred at 3.5 d in mucosa coinciding with downregulation of pathways that control apoptosis in both mucosa and whole gut. As expected, up-regulated DNA damage repair and cell cycle control signaling were mainly evident in the mucosa, i.e., the compartment where most DNA damage occurs. ATM, a key regulator of multiple signaling cascades in response to radiation-induced DNA strand breaks [24], [25], as well as ATM downstream targets Chk1 [26], Chk2 [27], and BRCA1 [28] were significantly up-regulated at this stage. The broad activation of these important DNA damage repairing pathways and down regulation of apoptosis pathway at 3.5 d coincided with sharply reduced TUNEL positive nuclei at this time.

It is interesting to note that up-regulation of pathways that control the coagulation system, lymphocyte apoptosis, tight junction, cell cycle control, and DNA damage repair was largely only found in the LCM-isolated mucosa but not in the whole gut extract. The sequential activation of these pathways after radiation exposure provides important information for understanding the mechanism of the injury. Thus, inclusion of the non-mucosal layers may seriously dilute the altered mucosal genes and disguise activation of relevant pathways.

A novel and unexpected finding was the marked increase of Casp14 and Eda2r expression, present in both mucosa and whole gut samples. The sharp and robust increase of these genes at the early time points suggests their important roles in the regulation of acute intestinal radiation injury.

Casp14 belongs to a conserved family of aspartate-specific proteinases. It is expressed in the suprabasal layers of the epidermis and is associated with protection against UVB-induced apoptosis and water loss [29], [30]. In the mouse mucosal LCM samples, radiation injury induced a 67-fold increase of Casp14 mRNA at 4 h, 33-fold at 24 h, and then rapidly returned to the baseline at 3.5 d. Immunohistochemistry verified this change at the protein level and also demonstrated that Casp14 was secreted from goblet cells that, as expected, mainly distributed on the surface of intestinal villi.

EDA2R is a type III transmembrane protein of the TNFR (tumor necrosis factor receptor) superfamily and has been identified as a p53 target which regulates p53-mediated anoikis [31]–[33]. Eda2r negatively regulates focal adhesion kinase (FAK), a central component of focal adhesion. As FAK functions as a regulator of epithelial cell survival and proliferation under conditions of mucosal injury, we speculate that the dramatic increase of Eda2r at 4 h and 24 h is associated with epithelial cell detachment and mucosal damage during acute intestinal radiation injury. The exact roles of Casp14 and/or Eda2r in the intestinal radiation response clearly need further study.

One concern of this study is the comparability of the gene expression profiles from LCM and whole gut. To address this problem, RNA samples for microarray processing were screened by checking their integrity and genomic contamination. The parallel expression pattern of Casp14 at both mucosa and the whole gut revealed the comparability of the expression profiles from LCM and whole gut. The analysis result of activated cellular injury, apoptosis, and DNA damage repair pathways in mucosa but not in the whole gut is consistent to the pattern of cellular injury demonstrated by TUNEL staining. Taken together, these data demonstrate the reliability of the analysis in this paper.

In conclusion, the present investigation revealed that alterations of many of the pathways known to undergo changes in response to radiation exposure are obscured if RNA extracts are obtained from whole gut samples as opposed to by LCM of the mucosa only. Hence, our data strongly suggest that RNA for analysis of pathways related to the coagulation system, lymphocyte apoptosis signaling, tight junction signaling, cell cycle control, and DNA damage repair signaling should be procured by LCM. This study also suggests that the newly identified genes Casp14 and Eda2r could provide novel research objectives and potential therapeutic targets to protect against or mitigate intestinal radiation injury. Future study will investigate RNA expression related to the position or differentiation state of the cells in the mucosa during intestinal radiation injury.
